# Improvement of classification accuracy in a phase-tagged steady-state visual evoked potential-based brain computer interface using multiclass support vector machine

**DOI:** 10.1186/1475-925X-12-46

**Published:** 2013-05-21

**Authors:** Chia-Lung Yeh, Po-Lei Lee, Wei-Ming Chen, Chun-Yen Chang, Yu-Te Wu, Gong-Yau Lan

**Affiliations:** 1 Department of Electrical Engineering, National Central University, Jhongli, Taiwan; 2 Laboratory of Integrated Brain Research, Department of Medical Research and Education, Taipei Veterans General Hospital, Taipei, Taiwan; 3 Institute of Brain Science, National Yang-Ming University, Taipei, Taiwan; 4 Institute of Computer Science and Information Engineering, National Ilan University, Ilan, Taiwan; 5 Science Education Center, National Taiwan Normal University, Taipei, Taiwan; 6 Department of Biomedical Imaging and Radiological Sciences, National Yang-Ming University, Taipei, Taiwan; 7 Department of Medical Image, Cheng Hsin General Hospital, Taipei, Taiwan

## Abstract

**Background:**

Brain computer interface (BCI) is an emerging technology for paralyzed patients to communicate with external environments. Among current BCIs, the steady-state visual evoked potential (SSVEP)-based BCI has drawn great attention due to its characteristics of easy preparation, high information transfer rate (ITR), high accuracy, and low cost. However, electroencephalogram (EEG) signals are electrophysiological responses reflecting the underlying neural activities which are dependent upon subject’s physiological states (e.g., emotion, attention, etc.) and usually variant among different individuals. The development of classification approaches to account for each individual’s difference in SSVEP is needed but was seldom reported.

**Methods:**

This paper presents a multiclass support vector machine (SVM)-based classification approach for gaze-target detections in a phase-tagged SSVEP-based BCI. In the training steps, the amplitude and phase features of SSVEP from off-line recordings were used to train a multiclass SVM for each subject. In the on-line application study, effective epochs which contained sufficient SSVEP information of gaze targets were first determined using Kolmogorov-Smirnov (K-S) test, and the amplitude and phase features of effective epochs were subsequently inputted to the multiclass SVM to recognize user’s gaze targets.

**Results:**

The on-line performance using the proposed approach has achieved high accuracy (89.88 ± 4.76%), fast responding time (effective epoch length = 1.13 ± 0.02 s), and the information transfer rate (ITR) was 50.91 ± 8.70 bits/min.

**Conclusions:**

The multiclass SVM-based classification approach has been successfully implemented to improve the classification accuracy in a phase-tagged SSVEP-based BCI. The present study has shown the multiclass SVM can be effectively adapted to each subject’s SSVEPs to discriminate SSVEP phase information from gazing at different gazed targets.

## Background

Brain computer interface (BCI) measures specific brain signals induced from elaborately designed tasks and translates the brain signals into control signals [1,2], which provides a promising channel for disabled patients to communicate with external environments. Among current noninvasive BCIs, the steady-state visual evoked potential (SSVEP)-based BCI has been widely mentioned due to its high information transfer rate (ITR) (~70 bits/min), little training, and high accuracy [3,4]. SSVEP is sinusoidal-type electroencephalogram (EEG) signal generated by the human visual cortex that is synchronized and phase-locked to user’s attended repetitive visual stimulation [5-7]. SSVEP-based BCIs place EEG electrodes in the vicinity of the occipital area and recognize user’s gazed target by analyzing the frequency or phase characteristics of the measured SSVEPs.

SSVEP-based BCIs can be at least divided into two categories in terms of coding techniques, one is the frequency-coded SSVEP-based BCI and the other is the phase-tagged SSVEP-based BCI [2,8-11]. Among current SSVEP-based BCIs, most SSVEP-based BCIs are frequency-coded systems. These frequency-coded systems utilize multi-frequency flickers to induce subject’s SSVEPs. Each visual target has a corresponding frequency which can be recognized on the estimated spectra, and the target number depends on how many frequencies are used for visual stimulation [8-10]. Nevertheless, due to the amplitude-frequency characteristic of SSVEP, some frequency ranges with poor SSVEP signal-to-noise ratio (SNR) should be excluded which usually results in a limited visual target number. In contrast, the phase-tagged system permits visual flickers to flash at the same frequency but being tagged with different phases. Accordingly, phase-tagged SSVEP systems have been developed to extend the available target number for SSVEP-based BCIs [2,4-12].

Several phase-tagged SSVEP-based BCIs have been developed. Lee et al. [2] implemented an eight-target system with flickering frequency set at 31.25 Hz, and averaged over an amount of wave cycles (sixty cycles) to achieve 95% accuracy. Jia et al. [12] developed a frequency and phase mixed coding technique, and found a phase mismatch between the phase difference of visual stimuli and the phase difference of measured SSVEP. Shyu et al. [13] designed a SSVEP-controlled hospital bed nursing system on FPGA platforms. Chang et al. [14] proposed stepping delay flickering sequence (SDFS) to achieve a phase-tagged SSVEP-based BCI independent of SSVEP phase calibration. Zhu et al. [4] accurately analyzed the phase synchrony between SSVEP and the flashing timing of visual stimulator measured from a photodiode. They concluded the variation of the phase difference between the flashing timing of visual stimulator and SSVEP, which might be caused by the phase deviations of visual stimulator. The variability is also called jitter, defined as the standard deviation of measured latencies [15]. Lopez-Gordo et al. [16] utilized phase-tagged amplitude modulation to drive four checkboard stimuli, and manifested the requisite of a calibration procedure for classifying the four targets. Though the SSVEP phase has been reported as an effective feature to implement SSVEP-based BCIs, nevertheless, the variation of the phase difference between visual stimuli and measured SSVEP, caused by the phase deviation of the visual stimulus [4,16,17], individual’s emotional condition [18], selective attention [19], nicotine [20], anticipatory anxiety [21], etc., could cause deterioration in the detected accuracy. Therefore, an effective phase classification approach to account for each individual’s phase difference of SSVEP is needed so that a reliable phase-tagged SSVEP-based BCI can be achieved.

This study presented a multiclass support vector machine (SVM)-based approach to cope with SSVEP phase variation in the use of a phase-tagged SSVEP-based BCI. SVM was originally introduced by Vapnik and its co-workers at AT&T Bell Laboratories [22-25]. It has shown its transcendent performance in many applications [26], such as object identification [27], speaker identification [28], text categorization [29], etc. Input data in SVM is mapped into high-dimensional feature space and a hyperplane is determined to completely separate the input vectors into non-overlapping classes [30-33]. In this paper, the amplitude and phase features of SSVEPs, collected in every 4 cycles at 20 Hz flickering frequency from gazing at different phase-tagged flickers, were extracted to train the multiclass SVM classifier. The trained multiclass SVM was then utilized to discriminate subject’s gazed targets. The multiclass SVM data classification using complex non-linear decision boundaries could be helpful to improve the statistical classification performance in phase-tagged SSVEP-based BCI design.

## Methods

### A. Subjects and EEG preparation

Twenty subjects (ten males and ten females), ages from 23 to 37 years old, were recruited to participate in this study. Each subject had corrected Snellen visual acuity of 6/6 or better, with no history of clinical visual disease. Table 1 lists the demographic data of the participants. The research was carried out in compliance with Helsinki declaration. All subjects gave informed consent, and the study was approved by the Ethics Committee of Institutional Review Board (IRB), Taipei Veterans General Hospital, Taiwan. All measurements were noninvasive and the subjects were free to withdraw at any time without any penalty. One unipolar EEG channel was used by attaching an electrode (Oz (+)) placed at Oz position with respect to a reference electrode (Oz (-)) placed at the right mastoid. The ground electrode was placed in the frontal position (Fpz). These EEG electrode placements were based on the international EEG 10–20 system [34]. Oz EEG signals were amplified, pre-filtered within 0.1 ~ 100 Hz (PowerLabTM, ADInstrument, Castle Hill, NSW, Australia), and digitized at 1 KHz (NI-USB 6259E, National Instrument) for further processes. The EEG recordings in all subjects were done by the same technician to minimize operation errors.

**Table 1:** The demographic data of the participants in the multiclass SVM study

**Number of subjects**	**20**
**Gender (number; %)**	
Male	10 (50%)
Female	10 (50%)
**Age (mean ± SD; range)**	
Years	29.85 ± 4.92 (23-37)

### B. System architecture and visual stimulus

Four light-emitting diodes (LED) (part number: LYBSB93W1303R012BP, LedTech Electronics Co., Taiwan; rise time < 50 μs; wavelength ranging from 400 to 700 nm), covered with thin white paper diffusers, were utilized as visual stimuli located on the four corners of a stimulus panel. A small cross was inlaid at the center of each visual stimulus to facilitate subject’s eye fixation. All LEDs were flickering at 20 Hz achieved by alternative ON and OFF states, in which the luminance of ON and OFF states were 168.7 candelas (cd/m^2^) and 8.1 cd/m^2^, respectively, measured by a luminance meter (LS-110; Konica Minolta Photo Imaging Inc., USA) resulting in Michelson contrast of 90.3%. The four LEDs were tagged with distinct phases equally distributed over a full 360° phase cycle. The designated phase for *i*^*th*^ LED is *θ*_*i*_ = (*i*-1) × 90° for *i* = 1 ~ 4, with corresponding latency of

(1) $$t_{i}=\frac{\theta_{i}}{360{}^{\circ}}\times T,$$

where *θ*_*i*_ is the phase delay, *t*_*i*_ is the latency for achieving *θ*_*i*_, *T* = 1 / *f*, and *f* = 20 Hz.

**Figure 1:**
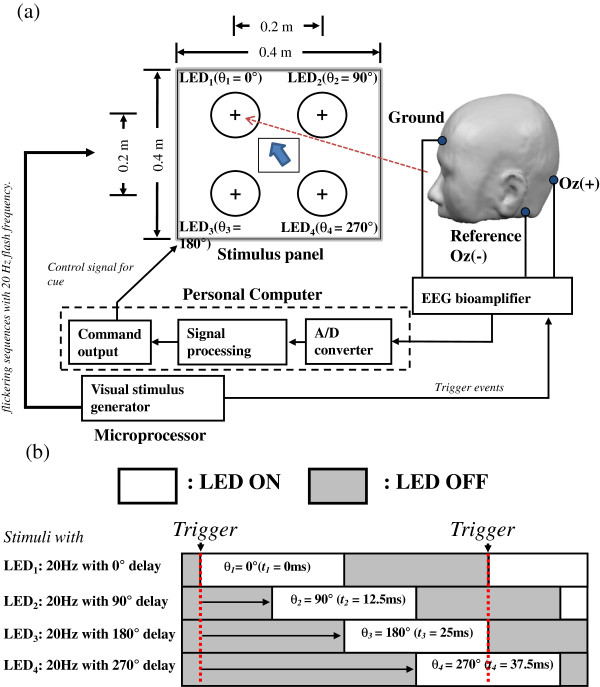
**Block diagram and flickering sequences of our phase-tagged SSVEP-based BCI system.** The system architecture of the system in (**a**). The four phase-tagged flickering sequences at 20 Hz, where the inter-stimulus phase delay was set at 90° (± 45° phase margin) in (**b**).

Figure 1a shows the system architecture of our phase-tagged SSVEP-based BCI. The stimulus panel was 60 cm in front of the participant. The four visual stimuli, tagged with 0°, 90°, 180° and 270°, were labeled as ‘LED_1_’, ‘LED_2_’, ‘LED_3_’, and ‘LED_4_’, respectively. The flickering sequences for driving the four visual stimuli are shown in Figure 1b. The phase lags of 0°, 90°, 180° and 270° are generated by manipulated time delays of 0 ms, 12.5 ms, 25 ms and 37.5 ms, respectively, in the four flickering sequences (see Equation (1)). The flickering sequences were generated by a microprocessor (C8051F120, Silicon Laboratories Inc., USA), programmed with Keil IDE software. The flash onsets (from OFF states to ON states) in the flickering sequences served as trigger events for the subsequent process.

### C. Experimental tasks

**Figure 2:**
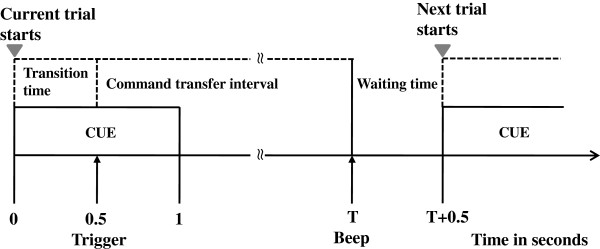
The experimental paradigm of the application study for each trial.

All subjects were requested to participate in a classification study and an application study. In the classification study, each subject was requested to gaze at each of the four visual stimuli for 60 seconds. After gazing at each visual stimulus, subjects were asked to take a 1 min rest. Another 60 seconds were also recorded from subject’s resting state while keeping his/her eyes open. We took the first halves (30 seconds) of the five recordings (i.e., four gaze recordings and one eye-opened resting-state (non-gaze) recording) for multiclass SVM training, and the other halves of the five recordings were used to test the feasibility of multiclass SVM in classifying different conditions. Since accurate classification usually depends on correct information of input data, statistical Z-test was applied to the input vectors (see below) obtained from each gaze condition of training data and those input vectors which rejected the null hypothesis (*p* < 0.05) were excluded from multiclass SVM training. In the application study, the subjects were instructed to shift their eyes to target the four visual stimuli for 80 trials (20 trials for each visual stimulus) in randomized order. Each trial contained the following steps: (1) An on-screen message (↖: LED_1_; ↗: LED_2_; ↙: LED_3_; ↘: LED_4_), located at the central position of the stimulus panel, was presented to instruct the subject to gaze at a designated visual stimulus; (2) The subject kept gazing at the designated visual stimulus until the subject received an auditory biofeedback. If the recognized gazed target was correct (i.e., the same as the one instructed by on-screen message), the auditory feedback would be a beep sound. Otherwise, a buzz sound would be generated to inform the subject of the wrong detection; (3) The subject perceived the auditory biofeedback, then shifted his/her gaze back to central position to prepare for next on-screen message. The experimental paradigm of the application study for each trial is shown in Figure 2.

### D. SSVEP signal processing

#### Extraction of SSVEP amplitude and phase features

Oz EEG signals were bandpass-filtered within 17–23 Hz to obtain SSVEP responses by means of applying a causal Butterworth filter (6th-order, IIR Butterworth filter). The frequency components of SSVEPs at 20 Hz were extracted in every 4 cycles using Fourier method with 75% overlapped sliding Hamming window. The frequency component at *i*^*th*^ time window can be expressed as:

(2) $$F_{i}=\frac{1}{K}\sum_{n=1}^{K}s_{i}\left[n\right]\cdot e^{\;-j2\pi \;\frac{f_{0}}{f_{s}}\;n},$$

in which *F*_*i*_ is the frequency component at *i*^*th*^ time window, *s*_*i*_[*n*] is the bandpass filtered data of *i*^*th*^ time window, *K* (*K* = 200) represents the sliding Hamming window length, and *f*_*s*_ (*f*_*s*_ = 1 KHz) and *f*_*0*_ (*f*_*0*_ = 20 Hz) are the sampling frequency and visual stimulus flickering frequency. The amplitude and phase features of frequency component *F*_*i*_ are arranged as an input vector, denoted as ***x***_*i*_, subjected to input points of multiclass SVM classification. The input vector ***x***_*i*_ is represented as

(3) $$x_{i}=\left[\begin{array}{c} \left|F_{i}\right|\\ ∠\left(F_{i}\right) \end{array}\right],$$

where | · | is the absolute value operator, ∠ (*F*_*i*_) = tan ^- 1^(Im{*F*_*i*_}/Re{*F*_*i*_}) is the phase angle of *F*_*i*_, and Im{*F*_*i*_} and Re{*F*_*i*_} are imaginary and real values of *F*_*i*_, respectively. In the classification study, one-minute Oz EEG signals were recorded for each condition. There were 2985 input vectors obtained from each subjects, including the four gaze and one resting-state (non-gaze) conditions. In the application study, the beginning 0.5 s transition time (10 input vectors) in each trial (see Figure 2) was excluded to avoid the contamination of eye-motion artifact.

#### Classification of SSVEP amplitude and phase features using multiclass SVM

We adopted “one-against-all” multiclass SVM which constructs one binary SVM for each class to distinguish samples of one class from samples of all remaining classes [26,35-37].

In this study, the first-half (30 s) input vectors obtained from each condition (597 input vectors for each condition; 2985 input vectors in total) in the classification study were used as input data to train the multiclass SVM for each subject. The trained multiclass SVM was used to evaluate the classification performance in the classification study and to detect the gazed targets in the application study. The amplitude and phase features of training data were arranged into a 2 × *N* (*N* = 2985) matrix for the multiclass SVM training. The multiclass SVM was constructed by a set of binary SVMs to map input data into output space. The classification of multiclass SVM was done by finding the class with maximum output among all binary SVMs. It is worthy to notice that the multiclass SVM should be retrained at the beginning of each session due to the consideration of inter-session variability in SSVEPs.

#### Determination of effective epoch for user’s gaze condition

**Figure 3:**
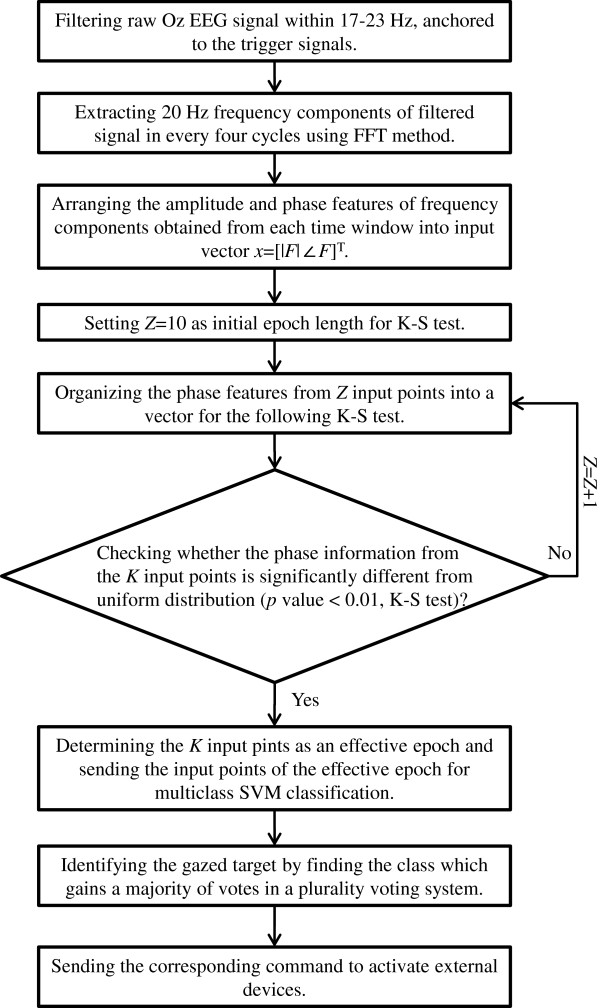
The signal processing flowchart of the proposed system.

Since SSVEP is time-locked and phase-locked signal contingent to the flickering timing of subject’s gazed target [34], user’s non-gaze condition will result in uncertainties in the detected SSVEP phases. SSVEPs induced from a phase-tagged visual stimulus have centralized phase distribution at the visual stimulation frequency, while resting-state Oz EEG signals usually have phase distribution, measured at the same frequency, randomly distributed over 0° to 360°. Accordingly, the discrepancy of phase distribution can be used to discriminate gaze condition from non-gaze condition in the use of a phase-tagged SSVEP-based BCI. In this paper, Kolmogorov-Smirnov (K-S) test was adopted to collect sufficient SSVEP phase information for gaze-target detections. K-S test is a non-parametric method to determine if two sample sets are different from each other [38,39]. Therefore, we reasonably assume the resting-state phases, measured at the flickering frequency of visual stimulus, of Oz EEG signals were uniformly distributed over a full cycle. The effectiveness of gaze-target information for a sample set can be examined by checking whether its phase distribution is away from uniform distribution or not. The length of an effective epoch for gaze-target detection was initially set at *Z* (*Z* = 10) input vectors and sent to K-S test to check its non-uniformness. If the data set could not reject the null hypothesis (i.e., *p* value > 0.01), one more input vector was added in the epoch. Once a data set passed K-S test, the data set was defined as an effective epoch. For each input vector in an effective epoch, a classification output was given by the trained multiclass SVM. The gazed target for each effective epoch was then identified using a plurality voting system by finding the class which gained a majority of votes. The overall flowchart of signal processing is shown in Figure 3.

## Results

**Figure 4:**
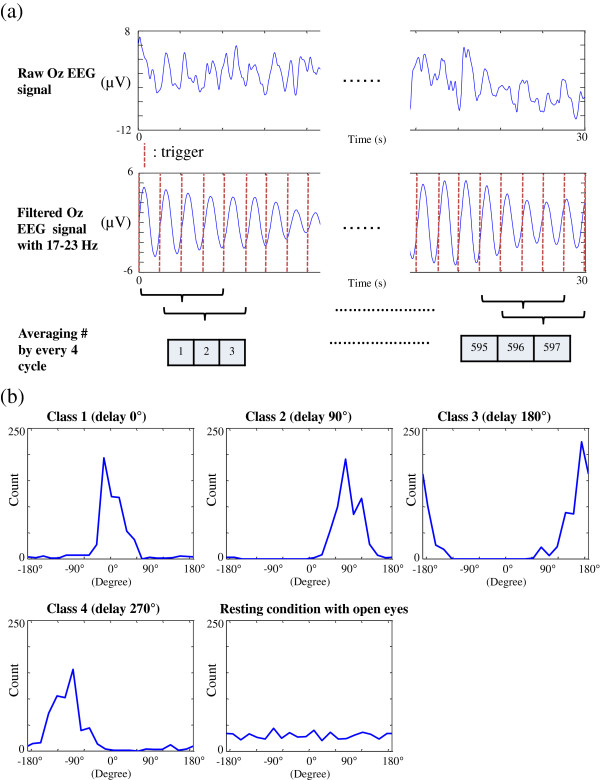
**The signal processing for extracting frequency components and phase distributions obtained from the five gaze conditions.** The signal processing for extracting amplitude and phase features in (**a**). Oz EEG signals were bandpass-filtered within 17–23 Hz to obtain SSVEP responses by means of applying a causal Butterworth filter (6th-order, IIR Butterworth filter). The frequency components of SSVEPs at 20 Hz were extracted in every 4 cycles using Fourier method with 75% overlapped sliding Hamming window. The difference of phase distributions obtained from the four gaze conditions (0°, 90°, 180°, 270°) and one resting-state (non-gaze) condition in subject 1 (**b**).

Figure 4a shows the signal processing for extracting amplitude and phase features in subject 1. The upper panel shows the raw Oz EEG signal from a 30-s recording and the lower panel presents the signal of raw Oz EEG signal filtered within 17–23 Hz. The flash onsets of LED flickering sequence, marked by red dashed vertical lines, were served as trigger events to segment the filtered EEG signal into cycles. The frequency components were extracted every 4 cycles with 75% overlapped sliding Hamming window, and the amplitude and phase features of frequency components were arranged into input vectors for multiclass SVM classifications. Figure 4b demonstrates the difference of phase distributions obtained from the four gaze conditions (0°, 90°, 180°, 270°) and one resting-state (non-gaze) condition in subject 1. Each phase distribution was the phase features of frequency components gathered from 30-s recording in different condition. It can be observed that the phase distribution recorded from the resting-state condition (right-lower panel in Figure 4b) is a uniform distribution which can be distinguished from other gaze conditions using K-S test.

**Figure 5:**
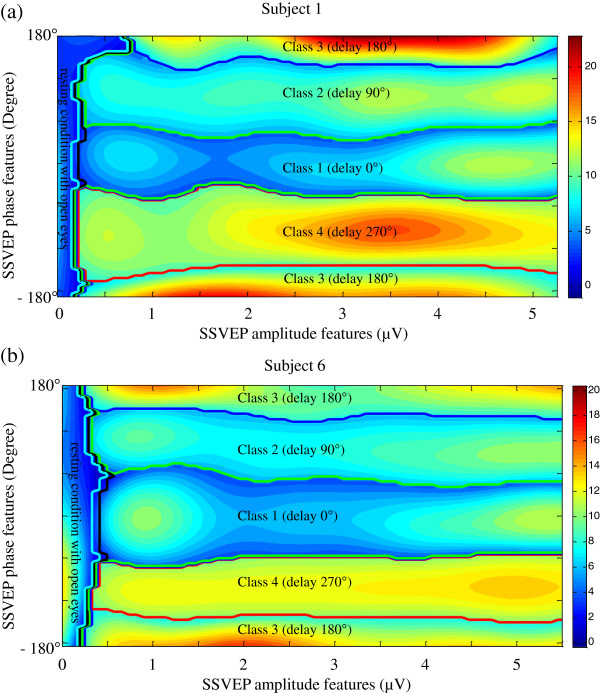
**The decision surfaces of the multiclass SVMs in classifying the five classes (four gaze and one resting-state (non-gaze) conditions) in subject 1 and 6.** The solid lines are the decision boundaries to separate each class from others. Colors code the outputs of decision function of the trained multiclass SVM.

In our classification study, one half of the recorded data was used for multiclass SVM training, and the other half was used for multiclass SVM testing. Figure 5 shows the decision surfaces of trained multiclass SVMs in classifying the five classes (four gaze and one resting-state (non-gaze) conditions) in subject 1 and 6. The solid lines are the decision boundaries to separate each class from others. Colors code the outputs of decision function of the trained multiclass SVM. The clear decision boundaries illustrate the feasibility of using the SSVEP amplitude and phase features in classifying different gaze conditions when operating a phase-tagged SSVEP-based BCI.

Table 2 presents the detected accuracies of multiclass SVM testing in the classification study. The averaged effective epoch lengths were from 0.59 s to 0.77 s; the averaged accuracies were from 87.18% to 97.70%; compared to the conventional epoch-average method with 14 cycles being averaged (detection interval = 0.7 s) [2], the detection accuracies were from 60.71% to 82.62% for 20 subjects. The overall accuracies were 93.49% vs. 70.70% (multiclass SVM testing vs. the conventional epoch-average method) which demonstrates the superiority (*p* < 0.01, Wilcoxon signed rank test) of using multiclass SVM in improving the SSVEP-based BCI performance.

**Table 2:** The detected accuracies of multiclass SVM testing and the conventional epoch-average method in the classification study

**Subject index**	**Multiclass SVM-based method**						**The conventional epoch-average method (detection interval = 0.7 sec)**
	**Accuracies for each class (Num**_**correct**_**/Num**_**total**_**)**				**Averaged accuracies (Num**_**correct**_**/Num**_**total**_**)**	**Averaged effective epoch lengths (s)**	
	**Class I**	**Class II**	**Class III**	**Class IV**			
1	97.62%	98.18%	94.23%	94.55%	96.08%	0.59	82.62%
	(41/42)	(54/55)	(49/52)	(52/55)	(196/204)		
2	97.96%	96.00%	91.30%	93.62%	94.79%	0.63	78.06%
	(48/49)	(48/50)	(42/46)	(44/47)	(182/192)		
3	97.56%	88.68%	90.38%	91.49%	91.71%	0.62	65.82%
	(40/41)	(47/53)	(47/52)	(43/47)	(177/193)		
4	95.56%	100%	94.34%	97.37%	96.79%	0.64	67.86%
	(43/45)	(51/51)	(50/53)	(37/38)	(181/187)		
5	94.59%	92.59%	91.38%	93.62%	92.86%	0.61	77.38%
	(35/37)	(50/54)	(53/58)	(44/47)	(182/196)		
6	94.87%	97.50%	95.45%	93.02%	95.18%	0.72	64.29%
	(37/39)	(39/40)	(42/44)	(40/43)	(158/166)		
7	91.43%	93.48%	97.73%	93.35%	94.64%	0.71	75.00%
	(32/35)	(43/46)	(43/44)	(41/43)	(159/168)		
8	94.59%	87.18%	82.50%	85.00	87.18%	0.77	80.40%
	(35/37)	(34/39)	(33/40)	(34/40)	(136/156)		
9	91.49%	96.15%	93.02%	97.83%	94.68%	0.64	70.92%
	(43/47)	(50/52)	(40/43)	(45/46)	(178/188)		
10	93.62%	88.64%	84.44%	92.86%	89.89%	0.67	68.39%
	(44/47)	(39/44)	(38/45)	(39/42)	(160/178)		
11	89.13%	90.48%	95.00%	87.50%	90.34%	0.68	66.33%
	(41/46)	(38/42)	(38/40)	(42/48)	(159/176)		
12	92.86%	91.67%	91.38%	90.20%	91.46%	0.60	71.94%
	(39/42)	(44/48)	(53/58)	(46/51)	(182/199)		
13	92.31%	93.18%	85.71%	94.00%	91.28%	0.62	73.98%
	(48/52)	(41/44)	(42/49)	(47/50)	(178/195)		
14	100%	91.49%	95.24%	87.50%	93.33%	0.67	65.31%
	(43/43)	(43/47)	(40/42)	(42/48)	(168/180)		
15	95.56%	89.80%	98.11%	100%	95.92%	0.61	63.78%
	(43/45)	(44/49)	(52/53)	(49/49)	(188/196)		
16	95.35%	98.00%	100%	97.50%	97.70%	0.69	68.88%
	(41/43)	(49/50)	(41/41)	(39/40)	(170/174)		
17	100%	91.30%	90.70%	95.12%	94.12%	0.71	74.49%
	(40/40)	(42/46)	(39/43)	(39/41)	(160/170)		
18	93.62%	100%	91.11%	87.76%	92.90%	0.66	72.96%
	(44/47)	(42/42)	(41/45)	(43/49)	(170/183)		
19	93.33%	92.45%	93.33%	97.67%	94.09%	0.65	60.71%
	(42/45)	(49/53)	(42/45)	(42/43)	(175/186)		
20	98.08%	91.11%	91.84%	97.78%	94.76%	0.63	64.80%
	(51/52)	(41/42)	(45/49)	(44/45)	(181/191)		
	Average (mean ± SD)				93.49 ± 2.58%	0.66 ± 0.05	70.70 ± 6.06%

To quantify the on-line performance of multiclass SVM, a command transfer rate (CTI) and an information transfer rate (ITR) are introduced in addition to the accuracy. The CTI is defined as the total experimental time (T_total_) divided by the number of total execution commands, and the ITR is defined as [1,40]:

(4) $$\frac{\mathrm{Bits}}{\mathrm{command}}=\log_{2}S+P\log_{2}P+\left(1-P\right)\log_{2}\left[\left(1-P\right)/\left(S-1\right)\right],$$

(5) $$\mathrm{ITR}=\frac{\mathrm{Bits}}{\mathrm{command}}\cdot \frac{60}{\mathrm{CTI}},$$

where *S* is the total number of visual flickers (*S* = 4) and *P* is the accuracy.

Table 3 shows the online results of execution time, accuracies, averaged effective epoch lengths and ITRs from twenty subjects in the application study. The accuracies were 90%, 97.5%, 91.25%, 83.75%, 93.75%, 96.25%, 88.75%, 95%, 88.75%, 86.25%, 92.5%, 91.25%, 87.5%, 85%, 96.25%, 81.25%, 93.75%, 90%, 86.25%, and 82.50%; the averaged effective epoch lengths were 1.12 ± 0.18, 1.11 ± 0.18, 1.10 ± 0.17, 1.16 ± 0.21, 1.15 ± 0.21, 1.09 ± 0.18, 1.10 ± 0.19, 1.16 ± 0.22, 1.17 ± 0.24, 1.12 ± 0.20, 1.10 ± 0.16, 1.11 ± 0.19, 1.14 ± 0.23, 1.13 ± 0.22, 1.11 ± 0.19, 1.13 ± 0.18, 1.19 ± 0.25, 1.20 ± 0.26, 1.16 ± 0.23 and 1.11 ± 0.17 s; the ITRs were 50.87, 66.72, 53.73, 39.73, 56.73, 64.68, 49.38, 59.14, 47.34, 44.49, 56.28, 53.27, 45.98, 42.31, 63.80, 37.07, 55.51, 48.41, 43.52, and 39.25 bits/min for subject 1 to subject 20, respectively. The averaged results (mean ± SD) of overall twenty subjects showed accuracy rate was 89.88 ± 4.76%, averaged effective epoch length was 1.13 ± 0.20 s and ITR was 50.91 ± 8.70 bits/min.

**Table 3:** The online results of execution time, accuracies, averaged effective epoch lengths and ITRs from twenty subjects in the application study

**Subject index**	**T**_**total **_**(s)**	**Accuracy (Num**_**correct**_**/Num**_**total**_**)**	**Averaged effective epoch lengths (s)**	**ITR (bits/min)**
1	129.50	90.00% (72/80)	1.12 ± 0.18	50.87
2	128.90	97.50% (78/80)	1.11 ± 0.18	66.72
3	128.05	91.25% (73/80)	1.10 ± 0.17	53.73
4	133.15	83.75% (67/80)	1.16 ± 0.21	39.73
5	132.30	93.75% (75/80)	1.15 ± 0.21	56.73
6	126.90	96.25% (77/80)	1.09 ± 0.18	64.68
7	127.75	88.75% (71/80)	1.10 ± 0.19	49.38
8	132.65	95.00% (76/80)	1.16 ± 0.22	59.14
9	133.25	88.75% (71/80)	1.17 ± 0.24	47.34
10	129.95	86.25% (69/80)	1.12 ± 0.20	44.49
11	127.65	92.50% (74/80)	1.10 ± 0.16	56.28
12	129.15	91.25% (73/80)	1.11 ± 0.19	53.27
13	131.35	87.50% (70/80)	1.14 ± 0.23	45.98
14	130.75	85.00% (68/80)	1.13 ± 0.22	42.31
15	128.65	96.25% (77/80)	1.11 ± 0.19	63.80
16	130.35	81.25% (65/80)	1.13 ± 0.18	37.07
17	135.20	93.75% (75/80)	1.19 ± 0.25	55.51
18	136.10	90.00% (72/80)	1.20 ± 0.26	48.41
19	132.85	86.25% (69/80)	1.16 ± 0.23	43.52
20	128.85	82.50% (66/80)	1.11 ± 0.17	39.25
Average (mean ± SD)	130.67 ± 2.60	89.88 ± 4.76%	1.13 ± 0.20	50.91 ± 8.70

## Discussion

Among the current assistive technologies, the BCI has drawn the greatest attention due to its independent of peripheral neuromuscular activities. A BCI recognizes the patterns of brain waves induced from elaborately designed task, and then translates the brain waves into control commands. However, since human brain is a complex system which usually exhibits remarkable inter-individual variability [12,41-44], therefore, choosing a robust classifier with flexibility to adapt inter-individual difference is crucial for an effective BCI. In this study, we adopted multiclass SVM to classify five conditions, including four gaze and one resting-state (non-gaze) conditions, in the use of a phase-tagged SSVEP-based BCI. The amplitude and phase information of SSVEPs were transformed into feature vectors for SVM training as well as classification. This is the first SVM study on the classification of phase-tagged SSVEP-based BCIs. The study results have shown the feasibility of the proposed system, which demonstrates an effective classifier could play an important role in designing a reliable BCI.

It has been mentioned several times in the literature that the amplitude of SSVEP can be used to discriminate the evoked SSVEP from background physiological activities. For example, Wu et al. [45] defined an SSVEP signal-to-noise ratio (SNR) in which comparing the SSVEP power divided by averaged power of the selected frequency window. Midderdorf et al. [46] applied an amplitude threshold for SSVEP to control a binary switch. However, it is difficult to judge subject’s gaze condition only from the information of SSVEP amplitude, owing to each subject has his/her own SSVEP amplitude-frequency characteristic which results in the SSVEP responses varying with different visual stimulation frequencies. Moreover, the inter-subject variation in background physiological activities also makes uncertainties in discriminating gaze condition from non-gaze condition if only SSVEP amplitude is used.

**Figure 6:**
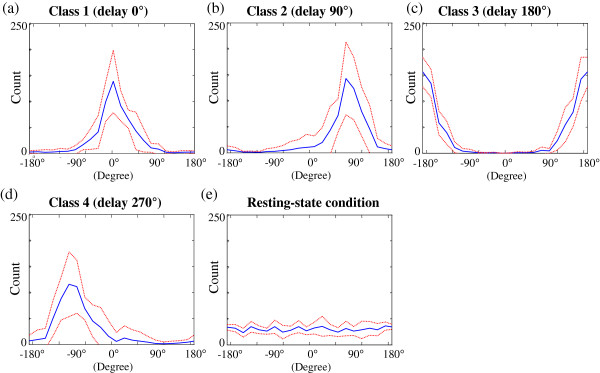
**The phase distribution of SSVEPs at 20 Hz obtained from the gaze and the resting-state (non-gaze) conditions in twenty subjects using Fourier method.** The phase distributions obtained from the four gaze conditions (**a** – **d**) showed significant differences with uniform distributions (*p* < 0.001, Kolmogorov-Smirnov (K-S) test), while the phase distribution checked by K-S test showed no statistical difference with uniform distribution (*p* > 0.05) in (**e)**.

Accordingly, in addition to SSVEP amplitude, we also took the information of SSVEP phase into account. Due to the advantages of time-locked and phase-locked characteristics of SSVEP, the SSVEPs generated from the subject’s gaze condition usually result in a centralized phase distribution. In contrast, the EEG phases obtained from subject’s resting-state (non-gaze) condition are irrelevant to flickering onsets of visual stimuli. Figure 6 shows the 20 Hz phases of Oz EEG signals obtained from the gaze and the resting-state (non-gaze) conditions in twenty subjects. The EEG phases were calibrated to the mean phase of SSVEP phases from gazing at LED_1_ (*θ*_1_ *=* 0°) in each subject for the purpose of cross-subject comparison. Figure 6a – e show the calibrated phases of Oz EEG signals at 20 Hz when subjects were gazing at LED_1_, LED_2,_ LED_3_, LED_4_, and resting-state (non-gaze) condition for thirty-second recordings (597 fast Fourier transform (FFT) time windows in each subject, FFT window length = 200 ms, window overlapping = 75%; twenty subjects pooled), respectively. The solid lines show the mean phases, while the dashed lines indicate the mean plus/minus the standard deviations (i.e., mean ± SD) over the twenty subjects. The phase distributions obtained from the four gaze conditions (Figure 6a – d) showed significant differences with uniform distributions (*p* < 0.001, K-S test). The phase distribution checked by K-S test showed no statistical difference with uniform distribution (*p* > 0.05) in Figure 6e. It echoes the decision surface of multiclass SVM results in Figure 5, in which the non-gaze class presented a uniform distribution spanning over 0 degree to 360 degree with low amplitude at stimulation frequency. It also endorses the feasibility of using phase distribution to discriminate gaze condition from non-gaze condition.

Since brain waves are dynamic activities which are contingent upon variations in a subject’s performance and state, linked to fluctuations in expectation, attention, arousal, and task strategy [47-52], the generation of command output from an EEG interval involving SSVEP-unrelated information may sometimes cause unexpected pitfalls in BCI operation. To prevent the outputs of trained multiclass SVM from oversensitivity to input data, the present study generated command outputs from effective epochs. An effective epoch was determined to define the length of input data, containing sufficient SSVEP information for a valid BCI output, by checking the phase distribution of input points using K-S test. The input vectors in a determined effective epoch were sequentially inputted into a trained multiclass SVM in each subject, and the multiclass SVM outputs generated from those input vectors were gathered to produce a valid output through a plurality voting process. The plurality voting process determined the valid output by finding the visual target which gained a majority of votes from the multiclass SVM outputs of all input points in the effective epoch, so that the accuracy and specificity of the proposed BCI could be improved.

In our previous publication [2], subject’s gazed target was identified using the phase feature of SSVEP only. Besides, the SSVEP was averaged over a large amount of epochs (~30 epochs) to increase SNR and the confidence of phase detection. The epoch-average process caused a long detection time (4.83 s/command) and resulted in the consequence of low ITR. In contrast, in this multiclass SVM study, both the amplitude and phase features of SSVEP were used and extracted in every 4 cycles. Input data (amplitude and phase features of SSVEP) were mapped into high-dimensional feature space by SVM kernel to achieve better classification. Owing to the input of dual features and the benefit of multiclass SVM in classification, the averaged detection time (T_total_/Num_total_) in our application study (see Table 3) is 1.63 s/command which is greatly shorter than the detection time in our previous study.

The present study focuses on testing the capability of using multiclass SVM to identify subject’s gazed targets by classifying amplitude and phase features of SSVEPs. However, according to some previous literatures, the phase of SSVEP can be influenced by the jitter of stimulator triggers [15,17], strategic planning, organized searching [53], memory load [54], emotional arousal [55], adaption of long-term stimulation [56], etc. Training a new classifier has to be learned at the beginning of each session or adopting on-line phase recalibration methods may be possible solutions to account for this phase drift problem in our current design. The issue of recalibrating on-line SSVEP phase can be referred to our previous publication [43] which utilized a biphasic stimulation approach to account for the phase drift of SSVEP during flicker stimulation. Our future work will combine this multiclass SVM method with on-line phase re-calibration technique to improve its applicability.

## Conclusions

In this paper, a multiclass SVM-based classification approach is proposed to discriminate subjects’ gazed targets for communication purposes. Subjects shifted their gazes at different visual stimuli, and the amplitude and phase information of the induced SSVEP were extracted for multiclass SVM to recognize the gazed targets. The salient features of the proposed system are: (1) the amplitude and phase features were used to classify four gaze and one resting-state (non-gaze) conditions; (2) the effective epoch was determined to define the length of input data, containing sufficient SSVEP information for a valid BCI output; (3) SVM outputs generated from input vectors of effective epochs were gathered to produce valid outputs through a plurality voting process. The system demonstrates its feasibility with ITR performance in healthy subjects. In future studies, the efficiency and reliability of this system for patients with motor neuron diseases should be further investigated.

## Competing interests

The authors declare that they have no competing interests.

## Authors’ contributions

CLY carried out the EEG data analysis by SVM and drafted the manuscript. WMC, CYC, YTW and GYL participated in the design of the study and performed the statistical analysis. PLL supervised the study, helped drafting and revising the manuscript. All authors read and approved the final manuscript.
